# Effect of functional badminton games on basic motor skills and sensory integration in 5–6-year-old preschool children: A randomised controlled trial

**DOI:** 10.1371/journal.pone.0335928

**Published:** 2025-11-14

**Authors:** Kang Zhang, Wei Zhang, Wenzhi Ding, Sha Cao

**Affiliations:** School of Physical Education, Sichuan Normal University, Chengdu, Sichuan, China; Universiti Kebangsaan Malaysia Faculty of Medicine: Hospital Canselor Tuanku Muhriz UKM, MALAYSIA

## Abstract

**Objective:**

This study aimed to develop functional badminton games for preschool children and investigate their efficacy in promoting basic motor skills and sensory integration.

**Methods:**

Sixty children aged 5–6 years were randomly assigned to an experimental group or a control group. The experimental group received a 12-week intervention consisting of functional badminton games, while the control group participated in regular physical activities. Measures of physical fitness, basic motor skills, and sensory integration were assessed at baseline and post-intervention.

**Results:**

The control group demonstrated significant improvements in the grip test, 15-meter steeplechase test, sit-and-reach test, standing long jump, locomotor subtest, ball skills subtest, and proprioception (**P* *< 0.01). The experimental group demonstrated significant enhancements in BMI, grip test, 15-meter steeplechase test, sit-and-reach test, standing long jump, vestibular function, tactile defensiveness, proprioception (**P* *< 0.01). All basic motor skill indicators in the experimental group exhibited significant improvements (*P* < 0.01) with large effect sizes (*d* > 0.80). Between-group comparisons revealed that the experimental group achieved significantly greater improvements in locomotor subtest, ball skills subtest, and vestibular function(*P* < 0.0167).

**Conclusion:**

Functional badminton games effectively enhance physical fitness, promote healthier BMI, and improve basic motor skills and sensory integration in preschool children. While regular physical activities also improve physical fitness, basic motor skills and proprioception, but they demonstrate limited effects on balance, BMI, vestibular function, tactile defensiveness, and learning ability. The findings indicate that functional badminton games are superior to conventional physical activities in enhancing basic motor skills and sensory integration, representing a valuable approach for promoting motor development in preschool children.

## 1 Introduction

Fundamental Movement Skills (FMS) comprise a broad spectrum of motor competencies that evolve from primitive reflexes [1].These skills establish the foundation for acquiring complex movement patterns and encompass both locomotor and manipulative abilities. Seefeldt et al. [2] introduced the concept of a proficiency ‘threshold’ in fundamental motor skill development, positing that achieving this threshold is essential for successful performance in daily activities. Substantial evidence has established strong positive correlations between fundamental motor skill development and multiple aspects of children’s physical and psychological well-being, including physical fitness, psychological adjustment, academic achievement, and executive function [3–6]. Sensory integration refers to the neurological process through which the brain receives, organizes, and interprets multisensory information from visual, auditory, tactile, vestibular, and proprioceptive channels to generate adaptive behavioral responses. Initially conceptualized by American psychologist Jean Ayres in the 1970s, this framework emphasizes the nervous system’s capacity to synthesize sensory information to support both motor skill development and cognitive processing [7]. Through complex neural networks, sensory receptors transmit processed information to the motor system, thereby facilitating the acquisition and execution of basic motor skills. Effective sensory integration provides precise neural guidance for motor actions, ensuring movement stability and accuracy.

The ‘Metaphoric Mountain Model’ identifies early childhood as a critical period for acquiring basic motor skills, during which children develop an extensive movement repertoire that forms the basis for later sports proficiency and skilled motor performance [8]. This developmental stage coincides with remarkable neuroplasticity, as the preschool-aged brain demonstrates exceptional adaptability and compensatory capacity, creating an optimal window for central nervous system maturation and sensory integration development [9–10]. Despite this developmental potential, alarming deficits in motor proficiency have been documented in preschool populations. ehan and Shams [11–12] reported substantially underdeveloped jumping, hopping, skipping, and object control skills, with approximately 84% of preschool children demonstrating significant difficulties in mastering overhand throwing techniques. Epidemiological studies indicate that 1.8–8% of children experience developmental challenges characterized by motor impairment, postural instability, and sensorimotor coordination deficits [13]. These concerning findings regarding developmental delays and sensory integration disorders have prompted international calls for evidence-based intervention strategies to address these pressing issues.

Embodied Cognitive Theory (ECT) provides a theoretical framework for understanding motor development, suggesting that fundamental motor abilities emerge through dynamic person-environment interactions rather than spontaneous maturation [14]. Physical activity serves as the mediating mechanism between the organism and its environment, enabling children to adapt to environmental demands. The sensory integration process plays a crucial role in this adaptive mechanism, facilitating the acquisition of basic motor skills. While empirical research and meta-analyses have demonstrated the efficacy of physical activity in promoting both basic motor skills and sensory integration in preschool children [15–16],existing interventions have predominantly focused on gymnastics and body intelligence games, leaving other movement forms underexplored. Badminton represents a promising but underinvestigated activity for promoting child development. As a whole-body sport characterized by movement complexity, environmental openness, and rich visual dynamics, badminton provides extensive opportunities for enhancing tactile and proprioceptive perception through striking actions and footwork patterns. However, the theoretical proposition that badminton may foster basic motor skills and sensory integration in preschool children remains largely unsupported by empirical evidence, with a notable scarcity of direct experimental verification.

Functional training offers a theoretically grounded approach to motor skill intervention. This systematic methodology, based on human movement chain theory, emphasizes movement quality and functionality by simulating task-specific patterns rather than isolating individual muscles [17–19]. By enhancing core stability, force transmission, and movement efficiency, functional training produces substantial benefits for children’s physical and psychological development. James and Costello [20–21] demonstrated that functional training reduces sports injury risk through improved body control, coordination, and trunk stability. Furthermore, Deng and Ketterer [22–23] documented that sensory integration and balance training effectively enhance attention, spatial perception, and problem-solving abilities across diverse populations. Collectively, these findings suggest that functional training may establish a foundation for lifelong physical activity participation and health through integrated physical-cognitive development.

Guided by these theoretical and empirical considerations, the present study integrated preschool children’s developmental characteristics with badminton’s unique features, including spatial cognition and tactile perception demands. We developed an innovative intervention program combining badminton with functional training theory, designing developmentally appropriate functional badminton games. This study aimed to investigate the effects of this intervention on physical fitness in 5–6-year-old children. We hypothesized that the functional badminton program would enhance physical fitness while promoting fundamental motor skill development and sensory integration. By providing empirical evidence, this research seeks to inform early intervention practices and raise awareness among parents and educators regarding the importance of fostering preschool children’s holistic development.

## 2 Materials and methods

### 2.1 Participants

**Fig 1:**
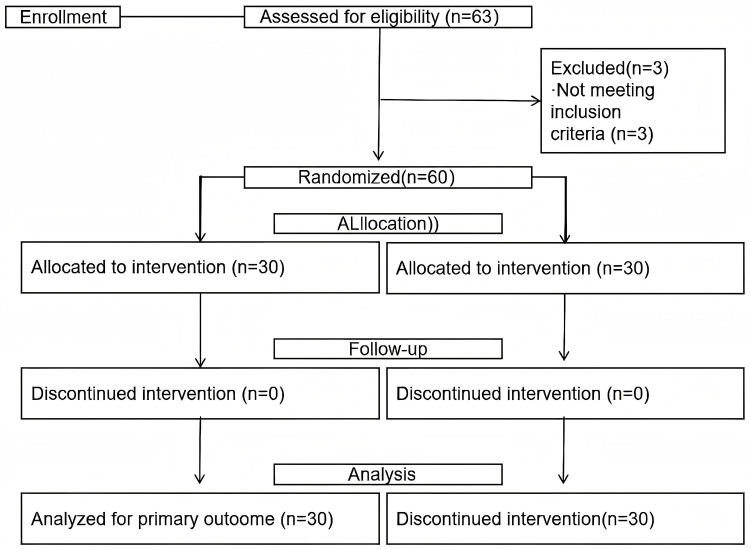
Participant flow diagram.

A priori power analysis was conducted using G*Power software with parameters set to effect size = 0.8, α = 0.05, and power = 0.9. This calculation was performed to determine the required sample size, which included 28 subjects each in the control and experimental groups. To account for potential attrition, sixty children aged 5–6 years were recruited based on inclusion criteria, with 30 participants each randomly assigned to the control and experimental groups. The inclusion criteria were: (1) no prior badminton training, (2) no participation in sports beyond the standard school physical education program, (3) good health without physical or mental illness, and (4) voluntary participation with expressed interest in badminton. The guardians of all the participants were fully informed of the experimental contents and signed written informed consent. The participant flow diagram is presented in Fig 1.

### 2.2 Research methods

#### 2.2.1 Experimental method.

Experimental Design: A 2 × 2 mixed-factor design was employed with group assignment (control vs. experimental) and time (pre-test vs. post-test) as independent variables. The dependent variables included basic motor skills, sensory integration, and physical fitness indicators.

Sample Selection: Cluster randomization was implemented wherein two senior kindergarten classes from the same kindergarten were randomly selected as the sample population. An independent researcher not involved in subsequent procedures allocated the two intact classes to experimental and control conditions using a random number table to ensure group comparability.

Intervention Implementation:The 12-week intervention was conducted from September 6 to December 6, 2023. The control group continued their regular kindergarten physical activities (including chasing, exercising, and climbing), while the experimental group received a functional badminton intervention grounded in functional training theory and tailored to preschool children’s developmental characteristics. The intervention program focused on practicing fundamental movements through structured badminton games (Table 1). Both groups completed a two-day baseline assessment of all outcome measures prior to intervention initiation.

**Table 1:** Intervention schedule.

Procedure	Time	Contents
Warm-up exercises	10-15 minutes	It is recommended that physical conditioning be initiated through the implementation of activities such as enjoyable warm-up drills and badminton pace exercises.
Core game	25-30 minutes	The programme is centred on functional badminton games, which are designed to develop basic motor skills and promote physical growth.
Physical challenge	5-10 minutes	The design of physical tasks in the form of competitive events, such as relay races, has been demonstrated to engender a sense of competition and cultivate team spirit.
Relaxation activities	5 minutes	It is recommended that physical recovery and emotional calm be promoted through the implementation of relaxing games or simple stretches.

A single-blind design was maintained throughout the study. Control group sessions were supervised by regular kindergarten teachers, while the experimental group intervention was delivered by two badminton-specialized postgraduate students. The experimental group participated in three 60-minute sessions weekly, each comprising: 10–15 minutes of warm-up exercises, 30–40 minutes of game practice, and 5 minutes of cool-down stretches (Table 1). The control group engaged in routine physical activities matching the frequency and duration of the experimental group. The study protocol received ethical approval from the Institutional Review Board (approval number: 2023LSTY001) and was conducted in accordance with the Declaration of Helsinki.

Intervention Plan: The functional badminton program was designed to enhance physical fitness, motor skills, and psychological development through gamified activities. Based on functional training theory and child development patterns [24,25], the program incorporated fundamental badminton movements (racket swinging, footwork, hitting, and chasing) and more complex actions (turning, jumping, sideways sliding). These movements were broken down into developmentally appropriate modules with progressive difficulty. The program incorporated engaging elements including role-playing (“Little Bird Fly Fly”), chase games (“Badminton Obstacle Run”), and competitive activities (“Badminton Relay Race”) to maintain participant engagement. The intervention progressed through three phases: initial (weeks 1–5) focused on equipment familiarity and basic movement mastery; intermediate (weeks 6–10) increased game difficulty and competitiveness to enhance movement fluency and stability; and final (weeks 11–12) emphasized skill application in complex contexts while fostering competition confidence, self-esteem, and teamwork.

Blinding Protocol: Assessment personnel remained blinded to group assignments throughout the study. Testers conducting motor skills and sensory integration evaluations had no involvement in intervention delivery and remained blinded during data collection and entry. Statistical analysis was performed by an independent statistician unaware of group codes until completion of all analyses. This separation of intervention, assessment, and analysis roles ensured objective outcome measurement while maintaining randomization concealment.

#### 2.2.2 Physical fitness measurement.

Physical fitness was evaluated according to the Chinese National Physical Fitness Standards (2023 Revision). Assessments included: body mass index (BMI), grip strength, standing long jump, double-legged jump, balance beam walking, sit-and-reach, and 15-meter steeplechase tests, providing comprehensive measures of muscle strength, coordination, flexibility, and agility.

#### 2.2.3 Basic motor skills measurement.

The Test of Gross Motor Development-Third Edition (TGMD-3) was used to assess motor proficiency [26]. The assessment comprises 13 items across two subtests: six locomotor skills (run, skip, slide, gallop, horizontal jump, hop) and seven object control skills (overhand throw, underhand throw, two-handed catch, one-hand stationary dribble, one-hand forehand strike, two-hand strike of stationary ball, kick of stationary ball). Each item was administered twice and scored against 3−5 criteria (1 point per criterion met). Raw scores, subtest scores, and total scores were computed following TGMD-3 guidelines.

#### 2.2.4 Sensory integration measurement.

The Children’s Sensory Integration Assessment Scale, a 58-item instrument specifically developed for Chinese preschool children, was used to identify sensory integration concerns. The scale assesses four domains: vestibular function, tactile defensiveness, proprioception, and learning ability, using a 5-point frequency scale (Never = 1 to Always = 5). Parents completed the scale based on their children’s typical behaviors. In this study, Cronbach’s alpha coefficients for each indicator were calculated using the formula $\alpha =\;\frac{K}{K-1}(1-\frac{{\mathrm{\backslash sumS}}_{i}^{2}}{S_{x}^{2}})$ (Table 2), where *K* represents the total number of measurement items, $S_{i}^{2}$ represents the variance of the first indicator dimension, and $S_{x}^{2}$ represents the total variance.

**Table 2:** Cronbach’s alpha coefficients for pre- and post-tests of sensory integration in the control and experimental groups.

Group	Experimental stage	Sensory integration	Vestibular function	Tactile defensiveness	Proprioception	Learning ability
control group	Pre-intervention	0.945	0.691	0.914	0.849	0.779
	Post-intervention	0.967	0.831	0.933	0.926	0.905
experimental group	Pre-intervention	0.927	0.768	0.862	0.830	0.764
	Post-intervention	0.952	0.830	0.906	0.876	0.821

#### 2.2.5 Mathematical statistics method.

Data were analyzed using SPSS Statistics (version 27.0). Normality of distribution was verified using P-P plots and Q-Q plots. For normally distributed data, within-group differences were analyzed using paired-sample t-tests, while between-group differences were assessed with independent-sample t-tests. The Bonferroni correction was applied for multiple comparisons, resulting in a significance threshold of *P* < 0.0167. Using the G*power effect size calculator, the effect sizes within and between groups were converted according to the formula $\text{Cohen}’d=\frac{{\text{M}}_{\text{1}}-{\text{M}}_{\text{2}}}{\sqrt{\frac{\text{SD}{\text{1}}^{\text{2}}-\text{SD}{\text{2}}^{\text{2}}}{\text{2}}}}$. The evaluation criteria were as follows: 0.20 < *d* ≤ 0.50 was considered a small effect, 0.50 < *d* ≤ 0.80 was considered a moderate effect, and *d* > 0.80 was considered a large effect size.

## 3 Results

### 3.1 Baseline homogeneity of measures

No significant between-group differences were observed at baseline for seven physical fitness indicators: BMI, grip test, 15-meter Steeplechase test, double-legged jump test, sit-and-reach test, balance beam walking test, and standing long jump test (all *P* > 0.05). With the exception of the two-hand strike of a stationary ball, ball skills subtest, and kicking a stationary ball, there were no significant differences in other basic motor skill indicators and sensory integration indicators (*P* > 0.05), confirming the homogeneity of the control and experimental groups prior to the intervention(Table 3).

**Table 3:** Between-group homogeneity test for each indicator in 5- to 6-year-olds before the experiment.

Domain	Test battery	Group (M ± SD)		t	*P*	Cohen’*d*
		Control group (n = 30)	Experimental group (n = 30)			
physiques	BMI (kg/m^2^)	13.94 ± 1.60	13.80 ± 1.17	0.334	0.741	0.09
	Grip test (kg)	5.42 ± 2.36	5.62 ± 1.52	−0.396	0.695	0.10
	15-meter steeplechase test (s)	6.88 ± 0.55	6.93 ± 0.85	−0.287	0.776	0.06
	Double legged jumps test (s)	4.95 ± 1.00	5.03 ± 0.98	−0.334	0.741	0.08
	sit-and-reach test (cm)	9.47 ± 5.05	9.37 ± 4.57	0.102	0.919	0.02
	Balance beam walking test (s)	6.41 ± 3.06	7.08 ± 3.45	−0.703	0.488	0.20
	standing long jump test(cm)	94.57 ± 15.27	93.60 ± 23.63	0.195	0.847	0.04
Basic Movement Skills	Run	6.93 ± 1.20	6.53 ± 1.50	1.007	0.322	0.29
	Skip	3.83 ± 1.68	4.13 ± 2.06	−0.674	0.506	0.15
	Slide	6.77 ± 2.12	6.10 ± 2.31	1.720	0.096	0.30
	Gallop	2.23 ± 2.37	1.57 ± 1.90	1.072	0.293	0.30
	Horizontal Jump	3.50 ± 1.99	3.80 ± 2.26	−0.564	0.577	0.14
	Hop	4.13 ± 1.71	3.37 ± 1.88	1.626	0.115	0.42
	**Locomotor Subtest**	27.40 ± 5.63	25.50 ± 6.52	1.138	0.265	0.31
	Overhand Throw	6.07 ± 1.59	5.87 ± 1.43	0.504	0.618	0.13
	Underhand Throw	5.37 ± 1.54	5.30 ± 1.36	0.158	0.876	0.04
	Two-hand Catch	3.60 ± 0.89	3.80 ± 1.27	−0.828	0.415	0.18
	One-hand Stationary Dribble	2.03 ± 1.60	1.87 ± 1.88	0.360	0.721	0.09
	Forehand Strike of a Ball	5.40 ± 2.12	4.83 ± 1.14	1.406	0.170	0.33
	Two-hand Strike of a Stationary Ball	7.72 ± 1.23	5.13 ± 1.69	5.757	<.001^**^	1.75
	Kick a Stationary Ball	7.27 ± 1.23	5.23 ± 1.92	3.052	0.005^**^	1.26
	**Ball Skills Subtest**	36.23 ± 4.24	32.03 ± 3.87	4.372	<.001^**^	1.03
sensory integration	Vestibular Function	57.57 ± 5.81	55.43 ± 6.87	1.503	0.144	0.33
	tactile defensiveness	85.77 ± 11.34	84.00 ± 11.13	1.072	0.292	0.15
	Proprioception	49.30 ± 6.58	47.70 ± 7.05	1.185	0.246	0.23
	Learning ability	32.53 ± 4.24	32.10 ± 4.52	0.419	0.678	0.09

** *P* < 0.01.

### 3.2 Changes in various indicators before and after the control group experiment

Following 12 weeks of regular kindergarten physical activity, the control group exhibited significant improvements in select physical fitness measures (Table 4). Specifically, the four performance indicators grip test, 15-meter steeplechase test, sit-and-reach test, and standing long jump test showed a significant increase (*P* < 0.01). In terms of effect size, the grip test and 15-meter Steeplechase test showed large effect sizes (*d* = 0.89, *d* = 1.00), whereas the sit-and-reach and standing long jump tests showed moderate effect sizes (*d* = 0.66 and *d* = 0.58). No other physical fitness indicators changed significantly.

**Table 4:** Changes in physical fitness pre-test to post-test scores of control group(N = 30).

Test battery	pre-testing	post-test	t	*P*	Cohen’*d*
BMI (kg/m^2^)	13.94 ± 1.60	13.67 ± 1.50	1.003	0.324	0.17
Grip test (kg)	5.42 ± 2.36	7.49 ± 2.26	−4.287	<.001^**^	0.89
15-meter steeplechase test (s)	6.88 ± 0.55	7.55 ± 0.77	−3.806	<.001^**^	1.00
Double legged jumps test (s)	4.95 ± 1.00	5.18 ± 0.84	−1.549	0.132	0.24
sit-and-reach test (cm)	9.38 ± 5.11	5.82 ± 5.61	3.343	0.002^**^	0.66
Balance beam walking test (s)	6.41 ± 3.06	6.56 ± 3.12	−0.261	0.796	0.04
standing long jump test(cm)	94.57 ± 15.27	103.37 ± 14.92	−3.420	0.002^**^	0.58

** *P* < 0.01

Significant pre- to post-intervention changes were also observed for basic motor skills (*P* < 0.01;Table 5). The locomotor subtest showed a large effect size (*d* = 2.23), whereas the ball skills subtest showed a medium effect size (*d* = 0.58). In addition, among the various indicators of locomotor subtest, theskip, hop, gallop, and horizontal jump showed significant improvements (*P* < 0.01). Furthermore, the gallop and horizontal jump tests showed large effect sizes (*d* = 1.35 and *d* = 1.47), whereas the skip and hop tests showed medium effect sizes (*d* = 0.44 and *d* = 0.5). Among the various indicators of physical control skills, the one-hand stationary dribble, overhand throw, Two-hand Catch, and Two-hand Strike of a Stationary Ball showed significant improvement (*P* < 0.01). Among these, the overhand throw, two-hand catch, one-hand stationary dribble, and two-hand strike of a stationary ball had large effect sizes (*d* = 0.97, *d* = 0.99, *d* = 1.52, and *d* = 0.95).

**Table 5:** Changes in scores on the pre-test-post-test of basic motor skills in the control group(N = 30).

Test battery	pre-testing	post-test	t	*P*	Cohen’*d*
Run	6.93 ± 1.20	7.43 ± 0.89	−1.945	0.062	0.47
Skip	3.83 ± 1.68	6.63 ± 1.77	−7.334	<.001^**^	0.44
Slide	6.76 ± 2.12	6.83 ± 1.64	−0.141	0.889	0.03
Gallop	2.23 ± 2.37	4.86 ± 1.40	−5.534	<.001^**^	1.35
Horizontal Jump	3.50 ± 1.99	6.0 ± 1.33	−5.839	<.001^**^	1.47
Hop	4.13 ± 1.71	6.60 ± 1.27	−6.771	<.001^**^	0.50
**Locomotor Subtest**	27.40 ± 5.63	38.36 ± 4.04	−10.338	<.001^**^	2.23
Overhand Throw	6.10 ± 1.64	7.39 ± 0.91	−3.178	0.004^**^	0.97
Underhand Throw	5.36 ± 1.54	6.20 ± 1.34	−2.506	0.018	0.58
Two-hand Catch	3.60 ± 0.89	4.36 ± 0.61	−4.678	<.001^**^	0.99
One-hand Stationary Dribble	2.03 ± 1.60	4.46 ± 1.59	−5.953	<.001^**^	1.52
Forehand Strike of a Ball	5.40 ± 2.12	4.30 ± 2.49	2.246	0.032	0.47
Two-hand Strike of a Stationary Ball	7.26 ± 1.22	5.66 ± 2.02	4.089	<.001^**^	0.95
Kick a Stationary Ball	6.50 ± 1.00	6.93 ± 0.94	−1.898	0.068	0.44
**Ball Skills Subtest**	36.33 ± 4.24	77.73 ± 5.98	−3.567	0.001^**^	0.58

** *P* < 0.01

Regarding sensory integration (Table 6), a significant improvement with a moderate effect size was observed solely for proprioception (t = −3.635, *P* < 0.01, *d* = 0.59). No other sensory integration indicators showed significant changes.

**Table 6:** Changes in pre-test-post-test scores of sensory integration of the control group(N = 30).

Test battery	pre-testing	post-test	t	*P*	Cohen’*d*
Vestibular Function	57.56 ± 5.81	58.96 ± 6.66	−1.177	0.249	0.22
Tactile Defensiveness	85.76 ± 11.34	89.43 ± 11.95	−1.854	0.074	0.31
Proprioception	49.30 ± 6.58	53.33 ± 6.86	−3.635	0.001^**^	0.59
Learning ability	32.53 ± 4.24	33.06 ± 5.14	−0.582	0.565	0.11

** *P* < 0.01

### 3.3 Changes in test scores before and after the experiment in the experimental group

In the experimental group, significant changes were observed across several physical indicators following the functional badminton intervention (Table 7). Five measures—BMI, grip test, 15-meter steeplechase test, sit-and-reach test, and standing long jump—improved significantly (*P* < 0.01). The grip test (*d* = 1.38) and the 15-meter Steeplechase test (*d* = 0.89) showed large effect sizes, whereas the sit-and-reach test (*d* = 0.67) and standing long jump test (*d* = 0.68) showed moderate effect sizes. BMI (*d* = 0.45) had a small effect on the results of this study.

**Table 7:** Changes in experimental group pre-test-post-test scores(N = 30).

Test battery	pre-testing	post-test	t	*P*	Cohen’*d*
BMI (kg/m^2^)	13.80 ± 1.17	13.20 ± 1.40	4.950	<.001^**^	0.45
Grip test (kg)	5.62 ± 1.52	8.30 ± 2.28	−6.534	<.001^**^	1.38
15-meter steeplechase test (s)	6.93 ± 0.85	7.89 ± 1.26	−3.610	0.001^**^	0.89
Double legged jumps test (s)	5.03 ± 0.98	5.06 ± 0.92	−0.222	0.826	0.03
sit-and-reach test (cm)	9.53 ± 4.57	6.03 ± 5.78	3.934	<.001^**^	0.67
Balance beam walking test (s)	6.70 ± 2.79	6.73 ± 3.34	0.542	0.592	0.009
standing long jump test(cm)	93.60 ± 23.63	107.97 ± 18.02	−4.335	<.001^**^	0.68

** *P* < 0.01

After the 12-week functional badminton intervention, basic motor skill indicators showed significant improvement (*P* < 0.01) with large effect sizes (all *d* > 0.80; Table 8).

**Table 8:** Changes in scores on pre-test to post-test of basic motor skills in the experimental group(N = 30).

Test battery	Pre-testing	Post-test	t	*P*	Cohen’*d*
Run	6.53 ± 1.50	7.73 ± 0.69	−4.207	<.001^**^	1.02
Skip	4.13 ± 2.06	7.23 ± 1.22	−8.255	<.001^**^	1.83
Slide	6.10 ± 2.31	7.8 ± 0.80	−4.233	<.001^**^	0.98
Gallop	1.57 ± 1.90	5.23 ± 1.30	−9.928	<.001^**^	2.24
Horizontal Jump	3.80 ± 2.26	7.0 ± 1.33	−6.869	<.001^**^	1.72
Hop	3.37 ± 1.88	6.47 ± 1.79	−6.553	<.001^**^	1.68
**Locomotor Subtest**	25.50 ± 6.52	41.47 ± 4.01	−14.181	<.001^**^	2.95
Overhand Throw	5.87 ± 1.43	7.93 ± 0.36	−7.628	<.001^**^	1.97
Underhand Throw	5.30 ± 1.36	7.47 ± 0.97	−9.406	<.001^**^	1.83
Two-hand Catch	3.80 ± 1.27	5.17 ± 1.05	−5.250	<.001^**^	1.17
One-hand Stationary Dribble	1.87 ± 1.88	5.6 ± 1.13	−9.517	<.001^**^	2.40
Forehand Strike of a Ball	4.83 ± 1.14	6.93 ± 1.33	−6.577	<.001^**^	1.28
Two-hand Strike of a Stationary Ball	5.13 ± 1.69	7.87 ± 1.77	−6.223	<.001^**^	1.58
Kick a Stationary Ball	5.23 ± 1.92	7.57 ± 0.77	−6.427	<.001^**^	1.59
**Ball Skills Subtest**	32.03 ± 3.87	48.53 ± 4.12	−16.918	<.001^**^	4.12

** *P* < 0.01

For sensory integration (Table 9), significant improvements (P < 0.01) were observed in vestibular function (moderate effect size, *d* = 0.79), tactile defensiveness (large effect size, *d* = 0.82), and proprioception (large effect size, *d* = 0.90).

**Table 9:** Changes in pre-test to post-test scores of sensory integration in experimental group.

Test battery	Pre-testing	Post-test	t	*P*	Cohen’*d*
Vestibular Function	55.43 ± 6.87	60.53 ± 5.93	−3.120	0.004^**^	0.79
Tactile Defensiveness	84.0 ± 11.13	92.4 ± 9.09	−3.476	0.002^**^	0.82
Proprioception	47.7 ± 7.05	53.50 ± 5.66	−4.000	<.001^**^	0.90
Learning Ability	32.10 ± 4.52	34.60 ± 3.89	−2.316	0.028	0.59

** *P* < 0.01

### 3.4 Intergroup comparison of intervention effects after the experiment

Compared with the control group, the experimental group did not show significant differences in the physical fitness test results. Only the grip test, walk the balance beam, and standing long jump tests showed an upward trend, but these differences were not significant (*P* > 0.05). In contrast, the experimental group achieved significantly higher scores than the control group on both the locomotor subtest (*P* < 0.01, *d* = 0.79) and ball skills subtest (*P* < 0.01, *d* = 2.18) of basic motor skill. Specifically, the experimental group scored higher than the control group in all indicators, including slide, horizontal jump, overhead throw, kick of a stationary ball, underhand throw, two-hand catch, forehand strike of a ball, one-hand stationary dribble, and two-hand strike of a stationary ball (all *P* < 0.01). Furthermore, the experimental group showed significantly better outcomes in vestibular function (*P* < 0.01) and tactile defensiveness (*P* < 0.0167) compared to the control group (Table 10).

**Table 10:** Differences in indicators between groups after the experiment.

Domain	Test battery	Control group	Experimental group	t	*P*	Cohen’*d*
physiques		13.67 ± 1.49	13.21 ± 1.41	−1.235	0.222	0.31
	grip test	7.49 ± 2.26	8.30 ± 2.28	1.378	0.174	0.35
	15-meter steeplechase test	7.55 ± 0.77	7.89 ± 1.26	1.276	0.207	0.32
	Double legged jumps	5.18 ± 0.83	5.06 ± 0.92	−0.522	0.604	0.13
	sit-up-and-bend	5.82 ± 5.61	6.03 ± 5.78	0.143	0.887	0.03
	walk the balance beam	6.56 ± 3.12	6.73 ± 3.34	0.203	0.840	0.05
	standing long jump	103.37 ± 14.92	107.97 ± 18.02	1.077	0.286	0.27
Basic Movement Skills	Run	7.34 ± 0.89	7.73 ± 0.69	1.450	0.153	0.48
	Skip	6.63 ± 1.77	7.23 ± 1.22	1.527	0.133	0.39
	Slide	6.83 ± 1.64	7.8 ± 0.80	2.896	0.006^**^	0.75
	Gallop	4.87 ± 1.40	5.23 ± 1.30	1.046	0.300	0.26
	Horizontal Jump	6.0 ± 1.36	7.03 ± 1.34	2.892	0.005^**^	0.76
	Hop	6.60 ± 1.27	6.47 ± 1.79	−0.332	0.741	0.08
	**Locomotor Subtest**	38.17 ± 4.3	41.47 ± 4.01	2.978	0.004^**^	0.79
	Overhand Throw	7.43 ± 0.89	7.93 ± 0.36	2.826	0.007^**^	0.73
	Underhand Throw	6.2 ± 1.34	7.47 ± 0.97	4.170	<.001^**^	1.08
	Two-hand Catch	4.37 ± 0.61	5.17 ± 1.05	3.593	<.001^**^	0.93
	One-hand Stationary Dribble	4.47 ± 1.59	5.6 ± 1.13	3.178	0.002^**^	0.81
	Forehand Strike of a Ball	4.30 ± 2.49	6.93 ± 1.33	5.098	<.001^**^	1.31
	Two-hand Strike of a Stationary Ball	5.67 ± 2.02	7.87 ± 1.77	4.476	<.001^**^	1.15
	Kick a Stationary Ball	6.93 ± 0.94	7.57 ± 0.77	2.841	0.006^**^	0.74
	**Ball Skills Subtest**	39.37 ± 4.27	48.53 ± 4.12	8.448	<.001^**^	2.18
sensory integration	Vestibular Function	58.97 ± 6.66	60.53 ± 5.93	4.592	<.001^**^	0.24
	tactile defensiveness	89.43 ± 11.95	92.4 ± 9.09	2.485	0.016^*^	0.27
	Proprioception	53.33 ± 6.86	53.50 ± 5.66	2.085	0.042	0.02
	Learning ability	33.07 ± 5.14	34.60 ± 3.89	1.881	0.065	0.33

* *P* < 0.0167 ** *P* < 0.01.

## 4 Discussion

### 4.1 Functional badminton games and physical fitness of preschool children

This study selected two senior kindergarten classes from a preschool to serve as the experimental and control groups. All participants were from the same preschool, between 5 and 6 years of age, and were highly homogeneous. This design effectively controlled for external variables, such as the educational environment, curriculum structure, and teacher qualifications. This facilitated the identification of the effects of the functional badminton interventions.

This study implemented a sports game framework that utilized key movement elements from badminton, including lateral movement, racket swing, footwork, and power generation. This study designed badminton game-based teaching content for preschool children based on functional training theory and adhered to the principles of educational value, enjoyment, scientific rigor, and content specificity. This enables the muscles involved in the movements to exercise naturally. The findings of this study demonstrate that both functional badminton games and conventional kindergarten physical activities are effective in enhancing physical fitness in preschool children. Significant improvements were observed in the grip, 15-meter Steeplechase, sit-and-reach, and standing long jump tests. There was a noticeable improvement in the performance of the double-legged jump test and walking on the balance beams. The findings of this study demonstrate that both functional badminton games and regular physical activities in kindergartens can effectively enhance preschool children’s upper and lower limb strength, explosive power, and flexibility in the same way. However, the impact of these factors on the balance is limited. In sensory integration tests, a significant improvement in vestibular function was observed in the experimental group. Currently, physical fitness tests primarily assess children’s balance by using balance beams. However, given the disparity in height between the beam and ground, children may experience psychological interference due to fear, which may not accurately reflect their actual balance ability. Future studies should incorporate objective measures, such as force platform measurements or wearable sensors, to evaluate static and dynamic balance capabilities more comprehensively. Latorre‐Román et al. [27] found that 10 weeks of regular physical activity enhanced jumping ability, speed, and endurance in children aged 3–6 years. Research has demonstrated that structured physical games based on schools and families can increase physical activity among preschool children, enhance their physical health, and promote the development of motor skills [28–29]. Notably, the BMI of preschool children who participated in the 12-week functional badminton game intervention showed a significant decrease. Conversely, no substantial changes were observed in the control group. The present study demonstrated that long-term participation in functional badminton games has the potential to reduce body mass index and improve the incidence of overweight and obesity in preschool children. However, the efficacy of regular physical activity in kindergartens is limited. Several studies conducted by BUMANMP, Wyszyńska J, and Huang W in various countries have established a strong correlation between BMI and intensity of physical activity [30–32]. Moderate- to high-intensity physical activity has been shown to be beneficial for physical fitness, whereas low-intensity physical activity has also been shown to confer certain health benefits, albeit relatively minor benefits. Consequently, we hypothesised that the effect of functional badminton games on improving preschool children’s physical fitness might be linked to the intensity of the exercise. However, there is a paucity of direct evidence to substantiate this hypothesis, and further research is required to validate these findings in humans.

### 4.2 Functional badminton games and basic motor skills in preschool children

The 12-week functional badminton game intervention significantly improved preschool children’s scores in various activities, including run, slide, gallop, hop, overhead and underhand throws, two-handed and one-handed catches, and kicking stationary ball. These findings suggest that badminton is a valuable tool to promote the development of locomotor and manipulative skills in preschool children. The fundamental principle of functional badminton games is the repetitive simulation of specific movements such as cross-steps, parallel turns, and swing strokes. This pedagogical approach is designed to ensure comprehensive activation of the muscles in the children’s upper limbs, lower limbs, and waist, thereby establishing a robust foundation for the development of basic motor skills. ALI et al. [33] found that physical activity based on fundamental movements can significantly improve all fundamental movement skill indicators in preschool children. Moghaddaszadeh et al. [34] summarized the effects of physical activity interventions on children’s basic motor skills and found that both sports-related and systematic physical activities can promote the development of gross motor skills in children. However, sports-related physical activities were the most effective, whereas game-based physical activities had no significant effect. Therefore, changes in the basic motor skills of preschool children may be related to the repetitive nature of the movements involved in functional badminton games. Long-term repetitive movement experiences deepen children’s cognitive understanding of movements and accelerate the motor learning of basic motor skills. The results of this study also showed that regular kindergarten physical activity can effectively improve performance in skip, hop, gallop, horizontal jump, overhand throw, two-hand catch, one-hand stationary dribble, and two-hand strike of a stationary ball. However, the effects were lower than those of functional badminton game interventions. Zhang D et al. [35] found that regular physical exercise has a certain promoting effect on the development of gross motor skills in preschool children, but there are individual cases where changes are not obvious and mastery is unstable; functional exercises have a significant effect on all indicators of gross motor skills in preschool children and are superior to regular physical exercise. This view is similar to that of the present study. Although regular physical activity in kindergartens can provide preschool children with certain opportunities for exercise, they often lack specificity and systematicity and fail to provide in-depth training and guidance on specific motor skills, thereby failing to fully meet the needs of preschool children in developing basic motor skills. Functional badminton games based on the functional training theory can effectively address this shortcoming and serve as an effective means of improving the basic motor skills of preschool children.

### 4.3 Functional badminton games and sensory integration in preschool children

The preschool stage is a window of opportunity for the development of motor skills, and a sensitive period for sensory integration. At this stage, basic motor skills and sensory integration overlapped in terms of time, indicating that basic motor skills and sensory integration tended to change synchronously. Previous studies have confirmed a strong positive correlation between basic motor skills in preschool children and static balance, dynamic balance, and proprioceptive ability [36–37]. The results of this study indicated that the experimental group exhibited significant changes in four indicators: vestibular function, tactile defensiveness, proprioception, and learning ability. In contrast, no significant changes were observed in the control group except for changes in proprioception. In addition, there were significant differences in the vestibular function and tactile defensiveness indicators between the control and experimental groups. These results suggest that functional badminton games have a significant potential to enhance preschool children’s sensory integration, whereas conventional kindergarten physical activities fall short of enhancing this ability. Fu et al. [38] found that a large muscle group exercise intervention had a significant positive correlation with vestibular function, tactile sensation, proprioception, and learning ability in special populations, such as autism. They concluded that the intervention design must reflect the respondencee and scientificity of the indicators of sensory integration. Chen D et al. [39] found that rhythmic physical activities with basic movement skills were better than general rhythmic physical activities in promoting gross motor development in children with sensory integration disorders, and concluded that repetition and variability in the process of practice were very important for the development of gross motor development in children with sensory integration disorders, and suggested that basic movement skills should be emphasised in rhythmic physical activities, and that repetition and variability should be used to encourage children to master basic movement patterns in a way that is consistent with the present study. The research ideas are consistent with the present study, suggesting that basic movement skills should be emphasized in rhythmic physical activities, and that repetition and variation should be used to promote children’s proficiency in basic movement patterns. The gradual increase in the number of lateral branches of neurones in the brain during early childhood and the enhancement of specific neural pathways with an increase in sensory integration information set the stage for the development of basic motor skills and sensory integration in children. According to existing research, various combinations of movements and game forms in functional badminton can promote the optimization of neuronesin the relevant brain areas through repeated movement stimulation, which, in turn, improves sensory integration. Previous studies have found that physical activity is strongly associated with the development of the structure and function of several brain areas [40]. Long-term physical activity increases overall brain volume in the gray and white matter regions, prefrontal and hippocampal volumes, and white matter integrity, and enhances functional connectivity between the default and frontal executive networks [41]. Shao et al. [42] found using a magnetic resonance imaging system that short-term badminton exercise increased the gray matter volume of brain functions related to visuomotor perception and increased the myelin thickness of fibre tracts, such as the posterior limb of the internal capsule and superior radiocorona in adults. These studies provide a basis for elucidating how functional badminton play can enhance basic motor skills and sensory integration in preschool children; however, targeted mechanistic research is lacking. In the future, the mechanism of action of functional badminton games in promoting the development of basic motor skills and sensory integration in preschool children can be further revealed from the perspective of neuroscience.

In summary, a 12-week functional badminton intervention effectively improved basic motor skills and sensory integration abilities in preschool children aged 5–6 years. This finding holds significant immediate practical value by providing empirical support for promoting structured physical activity programs in preschool settings. However, this study only conducted a post-test immediately after the intervention was concluded, failing to track the long-term retention of the effects of the intervention. Therefore, a core unanswered question is whether this significant immediate gain effect is temporary or translates into a stable, long-lasting developmental advantage. Wick K[43] and colleagues conducted strength training with 32 preschool children aged 4–6 years. After 10 weeks, significant changes in jumping ability were observed, accompanied by a trend toward improvedattentionaln capacity in preschool children. Zhang JY et al. [44] also found that 12 weeks of physical exercise could enhance the executive function of preschool children, suggesting that it could serve as an effective approach to promote the development of motor skills in preschool children. Mastering motor skills requires continuous practice and reinforcement. Without adequate environmental support and follow-up activities after the intervention ends, the benefits gained may gradually diminish over time. Conversely, if this intervention successfully sparks a strong interest in physical activity among children or introduces them to a virtuous cycle of sustained participation in sports, it may yield more profound effects. Future research should establish long-term follow-up assessment points, such as 3 months, 6 months, or even 1 year after intervention completion, to evaluate the long-term benefits. This will provide crucial scientific evidence for developing more sustainable physical activity promotion programs for children.

### 4.4 Limitations and prospects

First, this study recruited 60 children aged 5–6 years who met the experimental requirements. The samples were collected from Chengdu, China, and represent only a specific group of preschool children within a particular region, cultural context, and socioeconomic environment. This may have affected the generalizability of our results. Future research should expand the sample size to include more children from diverse geographical regions and backgrounds, and consider grouping children based on sex, age, physical characteristics, and sensory integration. By conducting large-scale studies, the generalizability of conclusions can be enhanced, providing stronger data support for policy formulation and practical implementation. Second, the control group participated in regular kindergarten physical activities and was not compared with other innovative exercise interventions. Therefore, the unique advantages of functional badminton games may not have been fully demonstrated by intervention results. In the future, other sports activities or innovative intervention methods can be introduced and compared with functional badminton games to comprehensively evaluate the intervention effects of different sports activities, providing diverse references for the design of preschool physical education courses. Additionally, the intervention content for the experimental group was implemented by the researchers, which may place higher demands on the technical capabilities of ordinary kindergarten teachers. To enhance the scalability and practical applicability of intervention measures, subsequent research should focus on training in-service kindergarten physical education teachers or relevant staff members to implement such interventions. In addition, we will further refine the instructional plan for functional badminton games based on kindergarten teachers’ professional backgrounds and teaching conditions. While ensuring the scientific validity and engaging nature of the activities, we will streamline operational procedures and reduce implementation costs, thereby enhancing the practicality and promotional value of this model.In later stages, the teaching plan for functional badminton games will be optimized based on the professional level of kindergarten teachers and actual teaching conditions, making it easier to operate and less costly while maintaining scientific accuracy and fun. Finally, significant differences were observed in motor skills and sensory integration among the children. Using a uniform intervention program may limit the exploration of the intervention’s effectiveness in different groups of children. In the future, it will be possible to design targeted, personalized intervention programs based on individual differences in preschool children’s motor skills and sensory integration and to develop personalized assessment tools and training modules to meet the diverse developmental needs of children.

## 5 Conclusion

This randomized controlled trial examined the effects of functional badminton games on basic motor skills and sensory integration in preschool children aged 5–6 years. Sixty children were randomly assigned to experimental and control groups. The experimental group received a 12-week intervention involving functional badminton games, while the control group engaged in regular physical activities. The results demonstrated that functional badminton games significantly enhanced physical fitness, maintained BMI, and improved both basic motor skills and sensory integration. Regular kindergarten physical activities also improved physical fitness and basic motor skills, including proprioception, but exerted limited effects on balance, BMI, vestibular function, tactile defensiveness, and learning ability. Functional badminton games were more effective than conventional kindergarten physical activities in enhancing basic motor skills and sensory integration thereby serving as a significant approach for promoting motor development in preschool children.

## Supporting information

S1 File Before. (XLSX)

S2 File Post. (XLSX)

S3 File Project proposal. (PDF)

S4 File CONSORT 2025 checklist. (DOCX)
