# Supplementary material for: Effect of functional badminton games on basic motor skills and sensory integration in 5–6-year-old preschool children: A randomised controlled trial
Source: PLoS One. 2025 Nov 14;20(11):e0335928. doi: 10.1371/journal.pone.0335928 (PMC12617838; doi:10.1371/journal.pone.0335928)
Supplement: S3 File — (PDF) [file pone.0335928.s003.pdf]

Project proposal

Study on the relationship between sensory integration ability, motor skills and body composition in children and functional training intervention

1. Research Background

In his report to the 20th National Congress of the Communist Party of China, President Xi Jinping emphasized the need to strengthen efforts in ensuring "early childhood education" and safeguarding children's physical and mental development. In 2016, the Ministry of Education's "Kindergarten Work Regulations" explicitly stated that kindergartens should prioritize promoting coordinated physical and mental development while cultivating outdoor activity interests[1]. Later that June, the State Council's "Healthy China 2030" Plan outlined initiatives to vigorously promote children's sports programs, aiming to foster healthy habits and lifelong fitness foundations[2]. In September 2019, the General Office of the State Council released the "Outline for Building a Sports Powerhouse," detailing youth sports development plans with particular emphasis on enhancing preschool physical education. The document improved policy frameworks and training systems for early childhood sports education[3]. In 2024, the Ministry of Education, National Health Commission, and National Administration of Disease Prevention and Control jointly issued guidelines requiring kindergartens and primary schools to ensure at least two hours of daily outdoor activities, including one hour dedicated to physical exercise[4]. These measures underscore the urgent imperative to advance comprehensive and high-quality health education for young children — a mission of profound significance that demands immediate attention.

Sensory Integration (SI) refers to the coordinated learning process between the human brain and body, which is crucial for lifelong learning[5]. Children's sensory integration ability serves as a vital foundation for promoting basic motor development and enhancing behavioral cognition in adolescents and children. Whether young children have impaired sensory integration directly impacts their physical health development[6]. Research on sensory integration dysfunction (SID) among preschoolers reveals severe issues in China. A study by Ren Guiying et al. found that Chinese preschoolers generally exhibit low levels of SI, with mild and severe dysfunctions occurring in 35.9% and 10.3% of normal school-age children in Beijing's urban areas[7]. In Changsha's urban area, the incidence rate of SID reached 35.03%, predominantly affecting gross muscle

coordination and balance functions[8]. Most affected children display symptoms including poor posture control, difficulty maintaining balance, classroom restlessness, attention deficits, weak muscle strength, uncoordinated movements, delayed language development, and cognitive impairments[9]. These challenges not only hinder physical growth but also negatively impact mental health. When sensory integration functions properly, children can process classroom information more efficiently. Visual discrimination and auditory attention are good, can recognize letters and numbers, understand the teacher's instructions, vestibular balance is conducive to maintaining sitting posture and concentration, proprioception is keen to help the development of hand fine motor skills and writing and painting ability, laying a good foundation for children's healthy growth.

The neuro-psychological development of children encompasses perception, motor skills, language acquisition, and psychological aspects such as memory, thinking, emotions, and personality traits, serving as crucial indicators of healthy growth[10]. The development of sensory integration stimulates neural growth in young children. During physical activities, infants simultaneously receive vestibular, proprioceptive, and tactile stimuli that enhance neuronal connections, strengthen interbrain communication, and promote cognitive development[11][12]. Neuro-psychological development is closely linked to nervous system maturation, particularly brain growth. Scientific research indicates the critical period for human brain development spans 0-6 years: by age 3, brain weight reaches 60% of adult levels, and by age 6, it reaches 90% of adult capacity[13]. The first three months post-birth mark the brain's rapid expansion phase, characterized by exponential neuron growth and coordinated development of sensory organs and the brain. From 3 to 12 months, motor skill-related brain regions develop rapidly. Ages 1-3 witness accelerated growth marked by explosive language acquisition, emotional awareness, and fine motor coordination. The critical refinement stage between ages 3-6 features deepening cognitive abilities, social skill development, and mature brain architecture[13]. The late preschool years (ages 5-6) represent a pivotal transition period for "kindergarten readiness," where sensory integration capabilities and

neuro-psychological development reach peak efficiency[14][15]. During this developmental stage, young children's brains demonstrate remarkable plasticity as various sensory pathways (including vision, hearing, touch, and proprioception) actively integrate. Well-developed sensory integration and neuropsychological growth enable children to perceive the world with greater precision and execute movements more fluidly. For instance, smooth writing requires coordinated proprioceptive and visual processing, while classroom concentration depends on auditory and vestibular system coordination. These foundational skills lay a solid groundwork for children's academic and daily life during their elementary school years.

Functional sports games are exercise methods that prioritize core stability while integrating children's motor skills, gross muscle development, proprioceptive training, and the coordination of strength with the nervous system. These activities effectively enhance physical fitness with strong entertainment value and health benefits[16]. Nowadays, functional sports games emphasize neural-muscular interactions through multidimensional joint training, promoting comprehensive development. They have been widely applied in competitive sports, athletic rehabilitation, and children's physical fitness programs[17]. For 5-6-year-olds, the primary neural signaling system dominates, characterized by active behavior, attention to novel stimuli, and imitation abilities. The secondary neural system is developing but remains underdeveloped, primarily showing limited language comprehension. Therefore, early childhood physical education should adopt game-based learning, skillfully combining physical exercises with specialized functional training. Innovative game designs like balance beam walking, obstacle jumping, and ball-pass relay incorporate fun competition elements, making them more engaging than traditional monotonous drills while ensuring sufficient training duration and frequency. Moreover, these games are designed according to sensory integration principles and child brain development patterns, precisely stimulating multiple sensory organs. For example, jumping games strengthen proprioception and vestibular sense, ball interactive games improve visual tracking and hand-eye coordination, crawling games exercise the whole body coordination and tactile perception. The multiple sensory input is

integrated by the brain to promote neural optimization, improve the state of sensory integration, and indirectly provide physiological support for neuropsychological maturity.

Previous studies have demonstrated that functional sports games can enhance young children's fundamental motor skills[18] and self-concept[19], while improving their physical fitness. However, there remains a lack of research on the impact of such games on sensory integration abilities and neuropsychological development in preschoolers. Current studies predominantly focus on how functional movement training affects sensory integration, yet few explore how these games specifically influence children's sensory processing capabilities given their unique physiological and psychological characteristics.

2. Research Objectives

Based on this study, we designed a functional sports game teaching program for children aged 5-6 by referencing the physical and mental development patterns of young children. Grounded in body-centered cognitive theory, human motor development theory, functional training models, and principles of preschool sports game design, our research investigates the dynamic interactions between functional sports games and sensory integration development and neuropsychological growth in typically developing children through instructional experimental interventions.

3. Research significance

Theoretical significance: This study takes functional sports games as the intervention means to explore the dynamic interaction between sensory integration ability and neuropsychological development of children aged 5-6. It not only provides a scientific basis for preschool education intervention, but also provides theoretical support for early prevention and intervention of developmental problems such as delayed motor development and sensory integration disorder in children.

Practical significance:(1) In view of the current lack of research on functional sports games for children aged 5-6, different types of functional sports games for children aged 5-6 are designed to enrich the content system of kindergarten sports games and provide reference for carrying out various forms

of sports games.

(2) Through 12 weeks of implementing functional sports games for young children, this study conducts comparative analysis on the impact of such games on sensory integration development and neuropsychological growth. The findings provide data support for early childhood physical education research, enabling preschool teachers to better select appropriate game content. This approach effectively enhances children's motor skill development, improves physical fitness, and promotes overall health.

4. Research content

(1) Design functional sports games suitable for children

This study first establishes a foundation through embodied cognition theory and human motor development theory, aiming to design physical education games for young children that foster their agency and experiential learning, thereby achieving dual educational objectives of "moral cultivation" and "emotional nurturing". Building on principles of functional training models and early childhood sports game design, we develop tailored physical activity programs for 5-6 year-old preschoolers based on comprehensive understanding of relevant curriculum standards.

(2) To verify the effects of functional sports games on the development of sensory integration and neuropsychological development in children aged 5-6 years

This paper intends to take functional sports games as the intervention means and select students from a kindergarten in Chengdu as the experimental objects. Through the comparative analysis before, during and after the experiment (the specific operation process is shown in 6.2.5), this study explores the influence of functional sports games on the development of sensory integration ability and neuropsychological development of children aged 5-6.

(3) Explore the dynamic interaction between the development of sensory integration ability and neuropsychological development

This study employed a functional sports game program as an intervention approach, selecting 5-6 year-old children from a kindergarten in Chengdu City as experimental subjects. Through comparative analysis of pre-experiment,

mid-experiment (6 weeks), post-experiment (12 weeks), and one-month follow-up test data after the intervention (detailed procedures see Section 6.2.5), we explored the dynamic interaction between functional sports games and sensory integration abilities and neuropsychological development in preschoolers aged 5-6 years.

5. Research subjects

In her study on the impact of functional sports games on young children's self-concept, scholar Li Suting selected 39 participants (20 in the experimental group and 19 in the control group). Gu Xinyu conducted a similar study to explore the effects of functional movement training on sensory integration abilities, selecting 31 students (16 in the experimental group and 15 in the control group). Wang Yingying investigated the influence of situational sports games on children's sensory integration and gross motor development, using 56 participants (28 in the experimental group and 28 in the control group). Building upon previous research and considering potential sample attrition, this study selected a senior kindergarten class in Longquanyi District, Chengdu City as the experimental site. Following recommendations from the kindergarten principal, teachers, and parents, we implemented random sampling to ensure a minimum sample size of 60 participants, which were then evenly divided into control and experimental groups.

Inclusion criteria: (1) No experience of sports training; (2) No participation in any other sports except school physical education courses; (3) Good health, no physical or mental illness; (4) Voluntary participation and strong interest in sports.

Exclusion criteria: (1) Have sports training experience; (2) Have physical or mental illness; (3) Have other aspects of sports training intervention.

6. Research methods

(1) Literature review method

Through studying embodied cognition theory, Introduction to Human Motor Development, Preschool Sports Game Curriculum Design, and Functional Training, this research establishes the suitability of functional sports games for

children aged 5-6. Using keywords such as functional training, functional sports games, sensory integration in early childhood, and neuropsychological development, we conducted literature searches across databases including China National Knowledge Infrastructure (CNKI), Sichuan Normal University Library, and Web of Science. The retrieved materials were systematically screened, organized, and analyzed to provide theoretical foundations and reference materials for this study.

(2) Game creation method

Based on embodied cognition theory and human movement development theory, combined with functional training mode and principles of preschool children's physical games, this paper designs functional physical games for 5-6 years old children, and takes this program as the intervention means

(3) Expert interview method

This study employs interviews conducted through face-to-face conversations, phone calls, and WeChat messaging to gather insights from early childhood sports specialists, preschool education faculty, functional training experts, and frontline educators. The research methodology focuses on clarifying key aspects including conceptual frameworks, implementation approaches, and curriculum design. By exploring the practical applicability and educational value of functional training for children, we aim to develop a tailored physical activity program for 5-6-year-olds. Building upon expert feedback, we will refine the experimental protocol to ensure its scientific rigor and operational feasibility.

(4) Experimental method

This study proposes to implement a functional sports game program as an experimental intervention for preschool children in the experimental class. The control group will continue regular kindergarten-based physical education activities, which are self-organized programs arranged according to national curriculum guidelines (with activity duration, frequency, and intensity aligning with those of the experimental group). Based on prior research and considering kindergarten schedules, both classes will have 60-minute PE sessions three times weekly for 12 weeks.

There were four tests in this experiment, which were baseline measurement

(before intervention), mid-intervention (week 6), late intervention (week 12), and follow-up test one month after intervention.

(5) Measurement method

1) Sensory integration ability assessment

① test tools

In alignment with the research objectives and content of this paper, and considering the experimental subjects involved, to establish consistent measurement standards that harmonize with previous studies while ensuring scientific rigor, we propose using the "Child Sensory Integration Development Assessment Scale" developed by Ren Guiying et al. for mainland China's children as the evaluation tool. This scale, validated through extensive testing, demonstrates practical significance with its straightforward question descriptions and broad coverage of children's daily experiences, which has gained strong parental recognition.

② Test content and standards

The Sensory Integration Rating Scale consists of five subscales: visual, auditory, vestibular, proprioceptive, and tactile. Each subscale evaluates responses on a 5-point scale (from "never" to "always"), with scores ranging from 1 to 5. Standardized T-scores are calculated based on these responses, where scores below 30 indicate severe sensory integration dysfunction (SID), while scores between 30 and 40 correspond to moderate or severe SID. The presence of any dysregulation in the sensory integration rating scale is considered a diagnosis of sensory integration dysfunction.

③ test mode

The scale was planned to be carried out in the form of online questionnaire, and the distribution and collection of the questionnaire were assisted by the kindergarten teachers of the experimental class and the control class in Chengdu. The scale was filled out truthfully by the parents of the children according to their recent actual situation and daily life performance.

2) Neuropsychological development assessment

①。 test tools

This study employs the "Neurodevelopmental Assessment Scale for Children

Aged 0-6" (hereafter "NDS") developed by Beijing Institute of Child Health as its assessment tool. The scale is divided into five dimensions: gross motor skills, fine motor skills, language and social behavior, and adaptive functioning. Through specialized testing instruments, it has been widely adopted in pediatric neurodevelopmental assessments.

②. Test content and standards

First, calculate the child's actual age in months and mark the primary measurement month. Then assess fine motor skills, social adaptation, language, social behavior, and gross motor skills (see appendix). If the scale shows an "R" character, it indicates this assessment can be referred to parents or kindergarten teachers. In the test, completed items are represented by o, while uncompleted ones are marked with x. Strictly follow the instructions when testing children. No one should interrupt the child to avoid prompting, suggesting, or guiding. Each item counts as 1 point if present; two items count as 0.5 points each. The intellectual age is calculated as 5 domain scores minus 5. Each child's evaluation is expressed as a developmental quotient (IQ), calculated as IQ = Intellectual Age / Actual Age × 100%. A developmental quotient above 130 is considered excellent, 110-130 is good, 80-110 is average, and 70-80 indicates a deficiency. Children with an overall developmental quotient <80 are diagnosed with psychomotor retardation; if any domain scores <80, it suggests delayed development in that area.

③. Measurement methods

This scale test plans to select 6 or more master's students from Sichuan Normal University whose research direction is preschool sports and health promotion, provide them with training guidance, and carry out practice in Qixia Kindergarten affiliated to Sichuan Normal University this semester, so as to ensure the accuracy of the results of the formal experimental test.

(6) Mathematical statistics method

The experimental data were entered into Excel tables, with non-compliant entries removed. Descriptive statistics were performed using SPSS26.0 to analyze sample characteristics and distribution patterns. Repeated-measurement ANOVA was conducted to compare inter-group and intra-group changes across

different time points. Pearson correlation and multiple linear regression analyses were employed to explore the dynamic interaction between sensory integration ability and neuropsychological development.

7. Experimental process

The experiment was divided into pre-experiment, preparation stage, experiment stage, post-experiment and one month after the experiment.

Before the experiment: The subjects were divided into experimental group and control group, and the pre-test of sensory integration ability and neuropsychological development of the experimental group and control group was carried out using "Children's Sensory Integration Ability Development Assessment Scale" and "0-6 years old children's Neuropsychological Development Scale", so as to obtain the pre-test data.

Preparatory Phase: During the two preparatory sessions before the formal experiment, students in the experimental group will receive instruction based on a pre-designed functional sports game program. This approach helps them adapt more quickly to the activity and better absorb new content, thereby avoiding excessive time spent correcting movements during the actual experiment. Such delays could otherwise lead to suboptimal outcomes, compromising both the efficiency and scientific rigor of the study.

Experimental Phase: The experimental group implemented instructional interventions through a specially designed functional sports game program, while the control group followed kindergarten curriculum content. Both groups engaged in fundamental outdoor activities including walking, running, jumping, and throwing games, with identical activity duration, frequency, and intensity to ensure comparable conditions. Around week 6 of instruction, both groups underwent standardized assessments. Data from these evaluations were analyzed against pre-instruction benchmarks to validate the scientific rigor and timeliness of the experiment.

After the experiment: After 12 weeks of functional sports game teaching, the sensory integration ability and neuropsychological development of children in the experimental class and the control class were measured and evaluated to obtain

the post-experiment data.

One month after the experiment: The children in the experimental group and the control group were tested again, and the data were compared and analyzed with that before teaching, so as to pay attention to the time dynamic relationship between functional sports games and sensory integration ability and neuropsychological development of 5-6 years old children.

8. Experimental quality control

(1) Excluding the interference of irrelevant variables to the experimental process, fully communicate the content, methods and testing process with kindergarten teachers and parents of children before the experiment, so as to avoid other related physical activities in the extracurricular exercise of the experimental group and the control group.

(2) Treat the subjects equally to maintain the effectiveness and stability of their learning and exercise.

(3) Before the test, solve the children's lack of understanding and timid mentality about the test items, conduct a comprehensive explanation and communication, and take two tests to get the highest score, so as to ensure that the scores of pre-test, middle test and post-test are of reference value.

9. Source of funds

1. Youth Fund Project of Humanities and Social Sciences Research, Ministry of Education (21YJC890044); 2. Youth Project of Philosophy and Social Science Fund of Sichuan Province (Project No.: SCJJ24ND289).
