# Supplementary material for: Effect of functional badminton games on basic motor skills and sensory integration in 5–6-year-old preschool children: A randomised controlled trial
Source: PLoS One. 2025 Nov 14;20(11):e0335928. doi: 10.1371/journal.pone.0335928 (PMC12617838; doi:10.1371/journal.pone.0335928)
Supplement: S4 File — (DOCX) [file pone.0335928.s004.docx]

| **\| CONSORT 2025 checklist of information to include when reporting a randomised trial** | | |
| --- | --- | --- |
| **Section/topic** | **No** | **CONSORT 2025 checklist item description** |
| Title and abstract | | |
| Title and structured abstract | 1a | Randomised trial |
|  | 1b | Objective: This study aimed to develop functional badminton games for preschool children and investigate their effects on basic motor skills and sensory integration.  Methods: Sixty children aged 5-6 years were divided into the experimental and control groups. The experimental group underwent 12 weeks of functional badminton game intervention, whereas the control group participated in regular physical activity. Physical fitness, fundamental motor skills, and sensory integration were measured before and after the intervention.  Results: The control group showed significant improvements in the grip test, 15-meter steeplechase test, sit-and-reach test, standing long jump, locomotor subtest, ball skills subtest, and proprioception (P < 0.01). The experimental group demonstrated significant enhancements in BMI, grip test, 15-meter steeplechase test, sit-and-reach test, standing long jump, vestibular function, tactile defensiveness, proprioception (P < 0.01). All basic motor skill indicators showed significant changes (P < 0.01) and large effect sizes (d > 0.80) in the experimental group. Compared with the control group, the experimental group exhibited more significant changes in the locomotor subtest, ball skills subtest, and vestibular function(P < 0.0167).  Conclusion: Functional badminton games effectively enhance physical fitness, maintain BMI, and improve basic motor skills and sensory integration in preschool children. Regular physical activities in kindergartens also enhance physical fitness and improve basic motor skills and proprioception, but have limitationssinn balance, BMI, vestibular function, tactile defensiveness, and learning ability. Functional badminton games are more effective than conventional kindergarten physical activities in improving basic motor skills and sensory integration, and serve as an important means of promoting motor development in preschool children. |
| Open science | | |
| Trial registration | 2 | No |
| Protocol and statistical analysis plan | 3 | S3 uploaded |
| Data sharing | 4 | Uploaded system (S1 and S2) |
| Funding and conflicts of interest | 5a | This research was supported by the Humanities and Social Sciences Fund of the Ministry of Education of China, "Research on the Impact of Characteristic Sunshine Sports Games on Motor Function of Preschool Children and the Relationship between Dose and Effect,” grant number 21YJC890044. |
|  | 5b | This study is devoid of any conflicts of interest. |
| Introduction | | |
| Background and rationale | 6 | Fundamental Movement Skills (FMS) are a wide range of movement skills developed from reflexes[1]. They form the basis for learning complex movement skills, and include motor and manipulative abilities. Seefeldt et al.[2] proposed that basic motor skills must be developed to a certain 'threshold’ to meet the demands of future daily activities. In recent years, many studies have confirmed that the development of basic motor skills is strongly positively correlated with children's physical and mental health, including physical fitness, psychological adjustment, improved academic performance, and development of executive function[4-6]. Sensory integration refers to the process by which the brain receives, integrates, and processes information from sensory channels, such as vision, hearing, touch, vestibular, and proprioception, and guides the body to respond adaptively to stimuli. This theory was proposed by the American psychologist Jean Ayres in the 1970s. At its core, it emphasizes the value of the nervous system's ability to coordinate multisensory information to form basic motor skills and cognition. The body's receptors organize, analyze, and process the information collected by the brain, and then transmit it through a complex neural network to the motor system to develop basic motor skills[7]. Good sensory integration provides accurate and unambiguous guidance for basic motor skills, ensuring stability and precision in movement execution.  The 'Metaphoric Mountain Model' model identifies the early years as the period of learning basic motor skills, where a diverse repertoire of motor skills is learned and built up, laying the foundation for later learning of complex sports and the flexible use of basic motor skills[8]. Simultaneously, the nervous system of young children is not yet fully developed, the brain is more plastic, and the compensatory capacity of brain cells is outstanding, making this period the best time for the maturation of the central nervous system and establishment of sensory integration[9-10]. However, a study by Behan and Shams found that children's jumping, hopping, skipping, and all object control skills were low, and as many as 84 per cent of young children had significant deficits in mastering overhand throwing motor skills, which manifested as a significant delay in the development of basic motor skills[11-12]. Research has indicated that 1.8 to 8 percent of children encounter developmental challenges characterized by impaired motor abilities, postural instability, and difficulties in sensorimotor coordination or motor learning[13]. Given the serious situation of developmental lag and sensory integration disorders in young children, there has been a common call from all sectors of the international community to seek and implement effective intervention strategies to address these issues.  According to the principles of Embodied Cognitive Theory (ECT), the acquisition of fundamental motor abilities in humans does not occur spontaneously; rather, it is derived from a dynamic interplay between the human organism and its physical environment[14]. Physical activity serves as a conduit between the body and environment, enabling children to adapt to dynamic changes in their environment. This process of sensory integration is a key aspect of motor development that facilitates the acquisition of fundamental motor skills in children. Empirical research and meta-analyses have demonstrated the efficacy of physical activity in promoting the development of fundamental motor skills and sensory integration in young children; however, the focus of this form of movement is predominantly on gymnastics and body intelligence games[15-16]. It is widely acknowledged that badminton is a whole-body sport characterized by the complexity and openness of its movement process and the richness of dynamic visual information. Participants have the opportunity to enhance the perceptual experience of tactile and proprioceptive sensations in striking action and foot movement; however, the causal links between badminton and the development of basic motor skills and sensory integration in young children can only be constructed at the theoretical levels. There is a paucity of direct evidence confirming the effectiveness of badminton in improving sensory integration and basic motor skills in young children.  Functional training is a systematic training method. It is based on the human movement chain theory, which emphasizes strengthening the quality and functionality of movements by simulating typical movements in daily activities and sports scenarios rather than exercising single or local muscles[17-19]. The improvement in trunk stability can be attributed to the core muscle strength. Furthermore, the promotion of force transmission, motor efficiency, and movement development are the key consequences of muscle strengthening. This type of training can substantially affect the physical and mental development of children. James and Costello concluded that functional training in children can reduce the risk of sports injuries by improving body control, coordination, and trunk stability through dynamic movements and static balance[20-21]. Furthermore, Deng and Ketterer used sensory integration and balance training to effectively enhance attention, spatial perception, and problem-solving abilities, which have the potential to improve in diverse populations[22-23]. Overall, functional training has the potential to provide children with a foundation for lifelong athleticism and health by rigorously integrating physical functions and cognitive development.  This study is based on the premise that the physical and mental developmental laws of young children should be integrated with the numerous characteristics of badminton, including but not limited to spatial cognition and tactile perception. The integration of badminton with functional training theory has been undertaken and functional badminton games that meet the developmental characteristics of young children have been designed have been designed. The present study aimed to explore the effects of functional badminton on the physical fitness of 5-6 year old children. We hypothesized that the game would enhance the children's physical fitness and promote the development of basic motor skills and sensory integration. This study was based on empirical evidence, and aimed to provide a scientific basis for early intervention in basic motor skills and sensory integration. This study also sought to help parents and educators to pay more attention to and promote the development of young children’s social skills. |
| Objectives | 7 | This study aimed to develop functional badminton games for young children and investigate their effects on basic motor skills and sensory integration. |
| **Methods** | | |
| Patient and public involvement | 8 | 60 children with 5-6 children were selected for this study based on the inclusion criteria, with 30 in each of the control and experimental groups. |
| Trial design | 9 | A two-factor mixed experimental design was adopted, with independent variable 1 being the group (control and experimental groups), independent variable 2 being time (pre-test and post-test), and the dependent variables being basic motor skills, sensory integration, and physical fitness indicators. |
| Changes to trial protocol | 10 | NO |
| Trial setting | 11 | This trial was completed at the kindergarten affiliated with Sichuan Normal University, Longquanyi District, Chengdu City, Sichuan Province, China. |
| Eligibility criteria | 12a | The inclusion criteria for the experimental subjects were as follows: (1) no prior training in badminton, (2) no participation in any sports other than the standard physical education program offered by the school, (3) good health and absence of physical or mental illness, and (4) voluntary participation and a strong interest in badminton. |
|  | 12b | The personnel responsible for implementing the interventions were all thoroughly trained before the formal experiment, ensuring they were well-versed in the requirements of each test. |
| Intervention and comparator | 13 | The experimental group used functional training as the basic theory, based on the physical development characteristics and patterns of young children. Functional badminton games were designed and implemented with the primary objective of practising basic movement (Table 1). Before the formal intervention, the control and experimental groups underwent a two-day assessment of basic motor skills, sensory integration, and various dimensions of physical fitness to obtain pre-intervention data. A single-blind experimental design was used for this intervention. The control group was supervised by full-time kindergarten teachers, whereas the experimental group implemented the intervention under the guidance of two postgraduate students specializing in badminton. Each exercise session lasted 60 min (10–15 min of warm-up exercises, 30–40 min of game exercises, and 5 min of stretching and relaxation) and was conducted thrice a week (Table 1). |
| Outcomes | 14 | The dependent variables were basic motor skills, sensory integration, and physical fitness indicators. The indicator tests are planned for two dates: September 6 and 7, 2023, and December 6 and 7, 2023. |
| Harms | 15 |  |
| Sample size | 16a | The parameters were set to 0.8, α=0.05, and power =0.9 using G* Power software. This calculation was performed to determine the required sample size, which was 28 subjects in each of the control and experimental groups. To prevent loss of sample size in the experiment, 60 children with 5-6 children were selected for this study based on the inclusion criteria, with 30 in each of the control and experimental groups. |
|  | 16b | NO |
| Randomisation: | | |
| Sequence generation | 17a | The subject matter leader designs methods for random sequence generation. |
|  | 17b | Cluster random sampling. Inter-group heterogeneity, And teachers are aware that groupings may introduce implementation bias and . |
| Allocation concealment mechanism | 18 | Use the Excel software to generate a random sequence, and then select the required number of classes based on these numbers. |
| Implementation | 19 | The randomised sequence was not known in advance by those responsible for recruitment and allocation.Participants were randomly allocated to either the experimental group (functional badminton game) or the control group (routine activities) to ensure baseline comparability between groups and to eliminate confounding bias. |
| Blinding | 20a | Outcome assessors and data analysts were blind after being assigned to the intervention. |
|  | 20b | By hiding grouping information, subjects are prevented from behavioural changes or subjective reporting bias due to knowledge of their grouping. |
| Statistical methods | 21a | Statistical analyses were performed using SPSS software (version 27.0). P-P and Q-Q plots were used to assess the normality of the data distribution for each indicator between the control and experimental groups. For data that met the criteria for normal distribution (including approximate normal distribution), the differences were tested using appropriate statistical methods. Paired-sample t-tests were used to analyze within-group differences in the pre- and post-test data between the control and experimental groups. Independent-sample t-tests were used to compare between-group differences between the control and experimental groups for the pre- and post-test data. Statistical significance was set at P < 0.05. Using the G*power effect size calculator, the effect sizes within and between groups were converted according to the formula $\text{Cohen’d=}\frac{\text{M}_{\text{1}}\text{−}\text{M}_{\text{2}}}{\sqrt{\frac{\text{SD}\text{1}^{\text{2}}\text{−SD}\text{2}^{\text{2}}}{\text{2}}}}$ . The evaluation criteria were as follows: 0.20 < d ≤ 0.50 was considered a small effect, 0.50 < d ≤ 0.80 was considered a moderate effect, and d > 0.80 was considered a large effect size. |
|  | 21b | All randomized participants。 |
|  | 21c | No data missing. |
|  | 21d | No additional analytical methods. |
| **Results** | | |
| Participant flow,including flow diagram | 22a | 60 children with 5-6 children were selected for this study based on the inclusion criteria, with 30 in each of the control and experimental groups. |
|  | 22b | Subjects who could not sustain participation or transfer during the process were removed. |
| Recruitment | 23a | Exercise intervention implementation: The intervention period was from September 6, 2023, to December 6, 2023, for a total of 12 weeks. |
|  | 23b | NO |
| Intervention and comparator delivery | 24a | The experimental group implemented the intervention under the guidance of two postgraduate students specializing in badminton. |
|  | 24b | Concomitant treatments that the groups did not receive during the trial. |
| Baseline data | 25 | Table 3. Between-group homogeneity test for each indicator in 5- to 6-year-olds before the experiment |
| Numbers analysed,outcomes and estimation | 26 | Table 4. Changes in physical fitness pre-test to post-test scores of control group（N=30）  Table 5. Changes in scores on the pre-test-post-test of basic motor skills in the control group（N=30）  Table 6. Changes in pre-test-post-test scores of sensory integration of the control group（N=30  Table 7. Changes in experimental group pre-test-post-test scores（N=30）  Table 8. Changes in scores on pre-test to post-test of basic motor skills in the experimental group（N=30）  Table 9. Changes in pre-test to post-test scores of sensory integration in experimental group  Table 10. Differences in indicators between groups after the experiment |
| Harms | 27 | NO |
| Ancillary analyses | 28 | NO |
| Discussion | | |
| Interpretation | 29 | The present study employed a sports game framework that utilized key movement elements from badminton, including lateral movement, racket swing, footwork, and power generation[27-29]. This study designed badminton game-based teaching content for young children based on functional training theory and adhered to the principles of educational value, enjoyment, scientific rigor, and content specificity. This enables the muscles involved in the movements to exercise naturally. The findings of this study demonstrate that both functional badminton games and conventional kindergarten physical activities are effective in enhancing physical fitness in young children. Significant improvements were observed in the grip, 15-meter Steeplechase, sit-and-reach, and standing long jump tests. There was a noticeable improvement in the performance of the double-legged jump test and walking on the balance beams. The findings of this study demonstrate that both functional badminton games and regular physical activities in kindergartens can effectively enhance young children's upper and lower limb strength, explosive power, and flexibility in the same way. However, the impact of these factors on the balance is limited. In sensory integration tests, a significant improvement in vestibular function was observed in the experimental group. Currently, physical fitness tests primarily assess children's balance by walking using balance beams. However, given the disparity in height between the beam and ground, children may experience psychological interference due to fear, which may not accurately reflect their actual balance abilities. Future studies should consider the use of professional equipment or different testing methods for follow-up verification. Latorre‐Román et al.[30] found that 10 weeks of regular physical activity enhanced jumping ability, speed, and endurance in children aged 3 to 6 years. Research has demonstrated that structured physical games based on schools and families can increase physical activity among young children, enhance their physical health, and promote the development of motor skills[31-32]. Notably, the BMI of preschool children who participated in a 12-week functional badminton game intervention showed a significant decrease. Conversely, no substantial changes were observed in the control group. The present study demonstrated that long-term participation in functional badminton games has the potential to reduce body mass index and improve the incidence of overweight and obesity in young children. However, the efficacy of regular physical activity in kindergartens is limited. Several studies conducted by BUMANMP, Wyszyńska J, and Huang W in various countries have established a strong correlation between BMI and intensity of physical activity[33-35]. Moderate- to high-intensity physical activity has been shown to be beneficial for physical fitness, whereas low-intensity physical activity has also been shown to confer certain health benefits, albeit relatively minor benefits. Consequently, we hypothesised that the effect of functional badminton games on improving young children's physical fitness might be linked to the intensity of the exercise. However, there is a paucity of direct evidence to substantiate this hypothesis, and further research is required to validate these findings in humans.  A 12-week functional badminton game intervention significantly improved young children's scores in various activities, including run, forward and slide, gallop, hop, overhead and underhand throws, two-handed and one-handed catches, and kicking stationary ball. These findings suggest that badminton is a valuable tool to promote the development of locomotor and manipulative skills in young children. The fundamental principle of functional badminton games is the repetitive simulation of specific movements such as cross-steps, parallel turns, and swing strokes. This pedagogical approach is designed to ensure comprehensive activation of the muscles in the children's upper limbs, lower limbs, and waist, thereby establishing a robust foundation for the development of fundamental motor skills. ALI et al.[36] found that physical activity based on fundamental movements can significantly improve all fundamental movement skill indicators in young children. Moghaddaszadeh et al. [37] summarized the effects of physical activity interventions on children's basic motor skills and found that both sports-related and systematic physical activities can promote the development of gross motor skills in children. However, sports-related physical activities are the most effective, while game-based physical activities have no significant effect. Therefore, changes in the basic motor skills of young children may be related to the repetitive nature of the movements involved in functional badminton games. Long-term repetitive movement experiences deepen children's cognitive understanding of movements and accelerate the motor learning of basic motor skills. The results of this study also showed that regular kindergarten physical activities can effectively improve performance in skip, hop, gallop, horizontal jump, one-handed ball tosses, underhand throws, overhand throws, two-handed catches, one-handed ball bounces, and two-handed hits against a stationary ball; however, the effects were lower than those of functional badminton game interventions. Zhang D et al.[38] found that regular physical exercise has a certain promoting effect on the development of gross motor skills in young children, but there are individual cases where changes are not obvious and mastery is unstable; functional exercises have a significant effect on all indicators of gross motor skills in young children and are superior to regular physical exercise. This view is similar to that of this study. Although regular physical activity in kindergartens can provide young children with certain opportunities for exercise, they often lack specificity and systematicity and fail to provide in-depth training and guidance on specific motor skills, thereby failing to fully meet the needs of young children in developing basic motor skills. Functional badminton games based on the functional training theory can effectively address this shortcoming and serve as an effective means of improving the basic motor skills of young children.  The preschool stage is a window of opportunity for the development of motor skills, and a sensitive period for sensory integration. At this stage, basic motor skills and sensory integration overlap in terms of time, indicating that basic motor skills and sensory integration tend to change synchronously. Previous studies have confirmed a strong positive correlation between basic motor skills in young children and static balance, dynamic balance, and proprioceptive ability[39-40]. The results of this study showed that the experimental group demonstrated significantly higher scores for all four indicators of vestibular function, tactile defence, proprioception, and learning ability, with particularly significant effects on the latter two indicators. In contrast, no significant changes were observed in the control group, except for proprioceptive changes in the ankles. In addition, there were significant differences in vestibular function and tactile and proprioceptive indicators between the control and experimental groups. These results suggest that functional badminton games have a significant potential to enhance young children's sensory integration, whereas conventional kindergarten physical activities fall short of enhancing this ability. Fu et al.[41] found that a large muscle group exercise intervention had a significant positive correlation with vestibular function, tactile sensation, proprioception, and learning ability in special populations such as autism. They concluded that the design of the intervention must reflect the correspondence and scientificity of the indicators of sensory integration. Chen D et al.[42] found that rhythmic physical activities with basic movement skills were better than general rhythmic physical activities in promoting gross motor development in children with sensory integration disorders, and concluded that repetition and variability in the process of practice were very important for the development of gross motor development in children with sensory integration disorders, and suggested that basic movement skills should be emphasised in rhythmic physical activities, and that repetition and variability should be used to encourage children to master basic movement patterns in a way that is consistent with the present study. The research ideas are consistent with the present study, suggesting that basic movement skills should be emphasized in rhythmic physical activities, and that repetition and variation should be used to promote children's proficiency in basic movement patterns. The gradual increase in the number of lateral branches of neurons in the brain during early childhood and enhancement of specific neural pathways with an increase in sensory integration information set the stage for the development of basic motor skills and sensory integration in children. According to existing research, various combinations of movements and game forms in functional badminton can promote the optimization of neurons in the relevant brain areas through repeated movement stimulation, which, in turn, improves sensory integration. Previous studies have found that physical activity is strongly associated with the development of the structure and function of several brain areas[43]. Long-term physical activity increases overall brain volume in the gray and white matter regions, prefrontal and hippocampal volumes, and white matter integrity, and enhances functional connectivity between the default and frontal executive networks[44]. Shao et al.[45] found using a magnetic resonance imaging system that short-term badminton exercise increased the gray matter volume of brain functions related to visuomotor perception and increased the myelin thickness of fibre tracts, such as the posterior limb of the internal capsule and superior radiocorona in adults. These studies provide a basis for elucidating how functional badminton play can enhance basic motor skills and sensory integration in young children; however, targeted mechanistic research is lacking. In the future, the mechanism of action of functional badminton games in promoting the development of basic motor skills and sensory integration in young children can be further revealed from the perspective of neuroscience. |
| Limitations | 30 | First, although this study recruited 60 children aged 5–6 years who met the experimental requirements, the sample size was relatively small, which may have affected the generalizability of the results. Future research should expand the sample size to include more children from diverse geographical regions and backgrounds, and consider grouping children based on sex, age, physical characteristics, and sensory integration. By conducting large-scale studies, the generalizability of conclusions can be enhanced, providing stronger data support for policy formulation and practical implementation. Second, the control group participated in regular kindergarten physical activities and was not compared to other innovative exercise interventions. Therefore, the unique advantages of functional badminton games may not have been fully demonstrated by the intervention results. In the future, other sports activities or innovative intervention methods can be introduced and compared with functional badminton games to comprehensively evaluate the intervention effects of different sports activities, providing diverse references for the design of preschool physical education courses. Furthermore, the intervention content in the experimental group was implemented by the researchers, which may pose technical challenges or resource constraints for ordinary kindergarten teachers, thereby affecting its practical feasibility. In later stages, the teaching plan for functional badminton games will be optimized based on the professional level of kindergarten teachers and actual teaching conditions, making it easier to operate and less costly while maintaining scientific accuracy and fun. Finally, significant differences were observed in motor skills and sensory integration among the children. Using a uniform intervention program may limit the exploration of the intervention’s effectiveness in different groups of children. In the future, it will be possible to design targeted, personalized intervention programs based on individual differences in young children's motor skills and sensory integration and to develop personalized assessment tools and training modules to meet the diverse developmental needs of children. |
